# Impact of Traditional Dance and Games on Cardiovascular Health: A Scoping Review of Outcomes Across Diverse Low- and Middle-Income Countries

**DOI:** 10.3390/ijerph22030440

**Published:** 2025-03-17

**Authors:** Adiele Dube, Ina Shaw, Musa L. Mathunjwa, Brandon S. Shaw

**Affiliations:** 1 School of Sport, Rehabilitation and Exercise Sciences, University of Essex, Colchester CO4 3SQ, UK; i.shaw@essex.ac.uk (I.S.); b.shaw@essex.ac.uk (B.S.S.); 2 Department of Human Movement Science, University of Zululand, KwaDlangezwa 3886, South Africa; mathunjwam@unizulu.ac.za

**Keywords:** aging, cardiovascular health, low-and middle-income countries, physical activity, interventions, traditional dance and games

## Abstract

In low- and middle-income countries (LMICs), where healthcare resources may be limited, the elderly are especially vulnerable to the adverse effects of cardiovascular diseases (CVDs). The aging population in these regions presents unique challenges, highlighting the urgent need for effective, accessible, and culturally appropriate interventions to address this cardiovascular health challenge in older adults. We aimed to evaluate the impact of traditional dance and games on cardiovascular health outcomes in LMICs through a scoping review of existing literature. This review followed the Preferred Reporting Items for Systematic Reviews and Meta-Analyses Extension for Scoping Reviews (PRISMA-ScR) guidelines. PubMed, Scopus, ProQuest, EBSCO, SPORT Discuss, Web of Science, and the grey literature were searched from 2000 to 20 September 2024. Two reviewers independently screened the titles, abstracts, and full texts and conducted data extraction. All conflicts were resolved with a third reviewer. A total of 3465 records were identified, of which 12 full-text articles were included in the review. The studies, five randomised clinical trials and seven non-randomised clinical trials, included varied age groups and populations including healthy, sedentary and obese participants. The interventions were traditional dance and games interventions with some extension to nutrition education. All the interventions were short term, with less than 6 months follow-up. Any traditional dance styles and games that involve physical performance can induce positive health outcomes. Undertaking traditional dance and games (TDGs) is equally effective on cardiovascular, functional and metabolic adaptations, leading to comparable improvements in older adults as for other forms of structured exercise. Collaboration of health practitioners, legislators, non-governmental agencies and local communities in LMICs in using TDGs may reduce the burden of CVDs.

## 1. Introduction

In 2013, the World Health Organization (WHO) aimed to reduce the burden of premature death caused by non-communicable diseases (NCDs) by 25% by the year 2025 [1]. In this regard, cardiovascular diseases (CVDs) are the leading cause of morbidity and mortality worldwide [1,2], with a particularly significant impact on the elderly population [3]. In 2021, 85% of the 17.9 million deaths from cardiovascular diseases were attributed to heart disease and stroke. As life expectancy increases globally, the prevalence of age-related cardiovascular conditions, such as hypertension, heart disease, and stroke, continues to rise [4], contributing to a growing public health burden [2]. In low- and middle-income countries (LMICs), where healthcare resources may be limited, the elderly are especially vulnerable to the adverse effects of CVDs due to factors such as inadequate healthcare access, poor nutrition, and a lack of preventive services [5,6]. It is known that at least three quarters of deaths from CVDs are reported in LMICs, where the poorest are most affected due to catastrophic spending on healthcare and high self-paying expenditure [2,7]. CVDs threaten the populations of LMICs and are driven by the acquisition of behavioural risks associated with urbanisation and globalisation, socio-economic and climatic changes, and an ageing demographic [8,9]. The aging population in these regions presents unique challenges, highlighting the urgent need for effective, accessible, and culturally appropriate interventions to address this public health challenge, cardiovascular health in older adults [10,11].

Maintaining physical activity (PA) in the latter stages of life is crucial for healthy ageing and quality of life (QoL), and the prevention and management of NCDs, such as CVDs [12]. This is because biological ageing and physical inactivity are both linked to significant declines in many physiological attributes, such as structural, physical, and functional deteriorations to the cardiovascular system [13]. In this regard, PA has primarily become a core target area for CVD prevention, and many countries world-wide have considered PA recommendations among their main components of healthy lifestyle guidelines. Despite such efforts, PA levels across age groups remains low, with one in four adults aged 65 years and older not meeting the WHO recommendations of 150 min of moderate-intensity PA or 75 min of vigorous-intensity PA and at least two or more days per week of major muscle-strengthening activities [14].

However, many LMICS, and especially rural communities in these countries, lack even the most basic healthcare access, including physical activity care, exacerbating health disparities, and leading to an increased risk for and manifestation of CVDs [7]. Immediate interventions are thus needed to address and curb the expansion of CVDs and ensure equitable healthcare in these LMICs [15].

Fortunately, many LMICs have existing traditional educational programs and structures instituting traditional dance, games and sport that enshrine their social, cultural, ideological, and socio-political aims [16,17]. Collaboration of healthcare systems with these indigenous communities may effectively promote a holistic approach to not only general well-being, but also the curbing of CVDs. Traditional dance and games are two forms of PA that are seen as enjoyable, easily accessible and lower cost, as well as requiring minimal equipment, and have previously been recommended for older adults [13,18,19]. Such traditional dance and games typically emphasise fun and enjoyment and are even performed competitively in some cultures [17,18]. From a lifestyle medicine aspect, traditional dance and games can be practised and performed individually or in groups with little equipment required for specific exercise environments, appealing to the different needs of participants, including people with physical and health conditions, and assisting in long-term exercise development [13,20,21]. In addition, the limited research on traditional dance and games has demonstrated that both can provide safe adjustable exercise prescription to suit a target population’s age, interests, and physical limitations [22,23]. Community-based CVD interventions are ideally suited for LMICs, and especially rural communities, to reduce the medical and national healthcare costs and burden exerted on the healthcare systems [11,24]. Problematically, research has demonstrated that, while many interventions including the community-led prevention strategies for NCDs have been conducted and implemented among individuals with existing CVD risk factors, their results are not yet unequivocal, calling for further research in this area [25]. Further, much of the research conducted on traditional dance and games has not focused on clinical or health-related outcomes related to CVD risk, especially in the elderly [24].

This study is necessary to influence public health policy and practice in LMICs by promoting culturally relevant, low-cost physical activities like traditional dance and games to improve cardiovascular health. These activities, deeply rooted in local cultures, could serve as cost-effective alternatives to conventional exercise programs, addressing the growing burden of CVD in resource-limited settings. By engaging communities with familiar, social activities, traditional dance and games could increase participation in health programs and promote preventive behaviours. The study could also inform future research and guide policymakers in integrating these practices into national health strategies, supporting health equity by providing accessible and culturally sensitive interventions for underserved populations.

Thus, the aim of this study was to evaluate the impact of traditional dance and games on cardiovascular health outcomes in LMICs through a scoping review of the existing literature. A scoping review is warranted to systematically map and analyse the existing evidence on the impact of traditional dance and games on cardiovascular health across diverse LMICs. Given the variability in cultural practices, health outcomes, and regional contexts, a scoping review will provide a comprehensive overview of the available literature, identify gaps in current research, and highlight the diversity of outcomes. This approach allows for a broad exploration of different interventions, ensuring that the findings are relevant to a wide range of LMIC settings, and can inform future research and public health policies effectively.

## 2. Methodology

This comparative scoping review followed the five steps supported by the Preferred Reporting Items for Systematic reviews and Meta-Analyses (PRISMA) Extension for scoping reviews (PRISMA-ScR) and recommendations from the Joanna Briggs Institute (JBI) [26,27]. The study protocol was registered on the Open Science Frame (OSF) (OSF.IO/8P2UH/). We combined findings from both qualitative and quantitative studies to address overlapping and or complementary information and identify gaps in the current literature.

Step 1: Identifying research question.

The main intention was to evaluate the scientific evidence on the impact of traditional dance and games on cardiovascular health across diverse LMICs. The following sub-research questions were identified:

- What is the evidence of the reported effects on cardiovascular health outcomes associated with traditional dance and games in diverse LMICs?
- What are quantitative and qualitative facets of these traditional dance and games across different demographic groups, settings and any specific population groups that show greater benefits from one of the interventions over the other and what are recommendations for future studies?

Step 2: Identifying relevant studies.

Search strategy:

Electronic sources from the following databases were searched: PubMed, Scopus, ProQuest, EBSCO, SPORT Discuss, and Web of Science. The grey literature was searched, and data were extracted using Google Scholar and Open Grey supplemented by the academic platforms ResearchGate and Academia.edu. Databases were searched from inception with the last search occurring on 20 September 2024. Articles were mainly searched using MESH terms “Dance OR Dancing OR Dance Therapy OR Traditional dance OR Cultural dance OR Dance effectiveness OR Dance interventions” AND “Traditional games OR Indigenous games OR Cultural games OR Ancestral games OR Heritage games OR Native games OR Nature games” AND “Cardiovascular health OR Cardio OR Cardiorespiratory fitness OR Oxygen consumption OR Physical health OR Cardiovascular endurance OR Heart health OR Quality of life”. Reference lists of the identified articles were also searched in case there were studies not found in the database search. The methodical quality of the identified and screened studies was assessed independently by two authors and any discrepancies were resolved by the third author. Table 1 below shows search parameters on various data sources. 

### Eligibility Criteria

Inclusion criteria: The articles in this review were full English original studies, reports, dissertations, and book chapters selected by identification of the search terms. Geographical restrictions were imposed to cater for those classified as LMICs according to the World Bank definitions and categorisations [28]. All types of traditional dance, games, and age groups were included. This review was not limited to randomised controlled trials and empirical evidence to find answers for the raised questions. This study included both RCTs and non-RCT studies to allow for a comprehensive overview of the available evidence and capture a wider range of study designs and real-world insights that RCTs alone may not fully represent. While RCTs are essential for assessing causality, non-RCT studies provided valuable information on the practical application, feasibility, and context of interventions, offering a broader understanding of their impact. This inclusive approach ensured that important findings were not overlooked due to methodological limitations and helped identify trends, gaps in the literature, and areas for future research.

Exclusion criteria: Systematic reviews and/or narrative reviews, virtual reality and video games, articles not in full text format and duplicated research were excluded from this study. Full text articles not in English were excluded, despite technology that permits translation and cross-lingual data extraction, the authors avoided translation to minimize distortion.

## 3. Results

### 3.1. Literature Search and Included Studies

**Figure 1:**
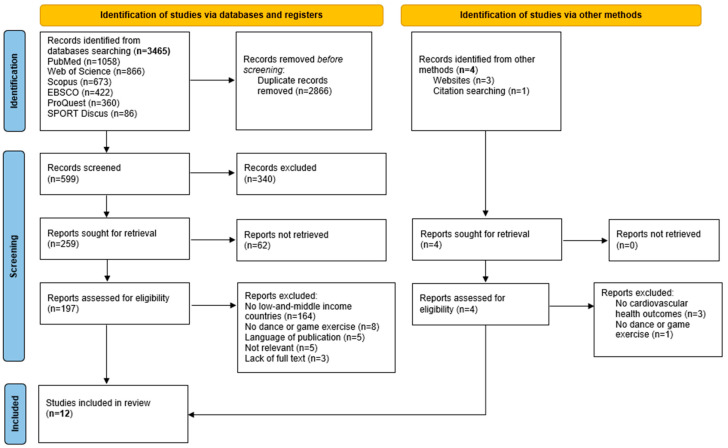
PRISMA flow chart of studies through searches.

The PRISMA flow chart (Figure 1) shows a total of 3465 potential studies identified through databases, registers and other methods searching. After removal of duplicates, 599 studies were screened by title and abstract, and 340 were excluded. Following the full text review of the remaining 197 articles and 4 articles identified manually, 12 studies that focused on the impact of traditional dance and games on cardiovascular health across diverse low- and middle-income countries with no comparison to other forms of exercises were included in this review.

### 3.2. Characteristics of Search and Included Studies

Table 2 and Table 3 show the impacts of dance and traditional games on cardiovascular health across diverse low- and middle-income countries. The twelve included studies comprised five RCTs and seven non-RCTs. Table 4 shows the countries investigated together with their Gross National Income per capita classification and Gross Domestic Product.

### 3.3. Characteristics of Participants

From the 12 studies included, a total of 1076 participants from LMICs were involved in dance and traditional games interventions, control groups or culturally related exercise. Of these, 301 participants were included in dance interventions and controls [13,29,30,31,32,33], 718 were fully engaged in traditional games [19,35,36,37], and 57 participants were engaged in both traditional dance and games but performed neither [24].

The participants were drawn from Brazil [13,24,37], China [30], Nigeria [31], Ethiopia [32], South Africa [33], Turkey [34], Indonesia [35,36], Malaysia [19], and Mozambique [24]. Their age range was from 9 to 80 years old. This diverse population included two studies with older adults [13,24], two studies with adults [29,34], four studies with children [19,33,35,37], one study with adolescents [32], two studies with no age mentioned, including women [30] and school students [36], and one study with older men [31]. Female gender was predominant across age groups [13,19,24,29,30,32,33,34,35,36,37], while the males were from a single study [31]. See Table 2 and Table 3.

## 4. Discussion

Our primary aim was to evaluate the existing evidence on the impact of traditional dance and games on cardiovascular health across diverse LMICs. There is promising preliminary evidence from randomized controlled trials, cross-sectional studies, and observational studies suggesting that people who engage in active cultural activities, including traditional dance and games, are likely to lead healthier lives across various stages of the lifespan, irrespective of their socioeconomic status [14,17,18,19,20,21,23]. To the best of our knowledge, this is the first scoping review explicitly evaluating the impact of traditional dance and games on cardiovascular health outcomes in LMICs, providing an opportunity for discussion and future research in this area and context. 

Various types of traditional dance have consistently been shown to improve cardiovascular fitness [13,29,31] and cardiorespiratory endurance [31] in healthy older adults and sedentary older adults. For instance, samba dance [29], simplified dance [30], and aerobic dance [31] notably increased VO_2_max and VO_2_peak after 8 and 12 weeks of intervention, respectively. All performed three sessions per week, lasting 45 to 60 min each. Ajala et al. [31], Wang et al. [30], and Malik et al. [19] suggested that dance and traditional games, which elicit moderate-intensity exercise at 60–70% of maximum heart rate achieved through progressive overload, are sufficient to improve the aerobic capacity of the elderly. For example, rhythmic movements involved in the Brazilian samba dance [29], Mozambique cultural and recreational activities [24] and the South African stick fighting game [33] are of high intensity, and thereby effectively improve blood circulation. Not only does this benefit blood circulation to muscles but also increases transportation of essential nutrients and oxygen to various organs and tissues. The concentration and motor control required during exercise might have immense positive impacts on brain function, resulting from increases in cerebral blood flow. These effects may be linked to mechanisms through which traditional games and dancing can enhance cardiovascular health [20]. In fact, the use of songs during traditional dance and games extrinsically motivates participants and positively influence cardiorespiratory responses.

Body composition changes were more pronounced in dance. Six interventions showed improvements in body composition, body fat percentage, lean body mass, and fat mass [13,29,30,31,33,35]. However, Ajala et al. [31] evaluated obese young men, who may have greater potential for body composition improvements. Samples of participants with different physical activity levels involved in traditional game interventions may have shown inconsistent statistical power to induce significant changes [33,35]. This could contribute to a gap in the available literature on traditional game interventions compared to other forms of exercise examining cardiovascular function outcomes.

Two traditional game intervention studies found no significant improvements in muscular function strength and endurance [24,33]. However, both studies did not specify whether muscle power was improved by traditional dance and game movements. Notably, the population studied by Rodrigues-Krause and colleagues [13], which consisted of sedentary older women, showed increases in lower body muscle power in dancing, with comparable results in body composition and cardiorespiratory fitness across various dance styles and walking. Despite these positive effects on muscular function and cardiovascular fitness, further research in more varied cohorts is needed to draw appropriate conclusions, particularly regarding traditional games for a broader population.

Currently, most studies included in the review have emerged from upper-middle-income countries (80%), with only twenty percent of studies from low-income countries. LMICs suffer the highest burden of CVDs [1,4]. Considering Africa’s unique cultural, ethnic, and socio-political factors, as well as its status as a lower-income region, it disproportionately bears the rising burden of CVDs [7,8,9,39,40]. It is vital for physical activity and exercise researchers to conduct more studies investigating CVDs in Africa, using community-based, local cultural activities as cost-effective alternatives to conventional exercise programs to promote management and prevention interventions and behaviours in resource-limited settings. With the existing literature focusing on the inclusion of traditional dance and games in physical education curricula [17,19], shifting priorities from the classroom to research funding, infrastructure, and relevant skills development could help bridge the literature gap in these countries and positively contribute to an active lifestyle, especially among adults.

The included studies show that metabolic measures positively responded to dance and traditional games. Notably, there is a balance of four RCTs and four non-RCTs for metabolic outcomes. Remarkable improvements were observed in blood biomarkers—total cholesterol, high-density lipoprotein, and markers of oxidative stress [24,30,32,34], as well as surrogates—blood pressure and low-density lipoprotein cholesterol [24,32,36,37]. Two RCT studies concurred with a non-RCT study that total cholesterol and triglycerides were significantly reduced after 12 weeks of simplified dance and culturally relevant activities [24,30,36]. In fact, high volumes of physical activity and/exercise and subsequent total energy expenditure increased significantly, improving cardiorespiratory fitness [13,24]. In an observational study with a large sample of 600 participants, traditional games elicited sufficient moderate-to-vigorous physical activity (MVPA), based on metabolic equivalents (METs) and counts per minute [19]. Through long-term adherence to sufficient MPVA, enhanced cardiorespiratory fitness has been linked with reductions in cardiovascular risk factors. Blood pressure, a surrogate endpoint metabolic outcome, responded to traditional dance and games interventions after 12 weeks [24,32]. Indeed, moderate to vigorous traditional dance and games may be linked to reduced risks of CVD mortality [19,34,41], which might lead to lifelong adherence to exercise, considering that these activities are available in communities at little or no cost and can be passed down through generations. Further research that provides follow-up and allows for systematic and meta-analytic reviews of effectiveness would generate average results from several trials.

One of our unique findings is that the experiments in all the included studies were short-term, comprising no more than 12 weeks with no follow-up of participants. Due to the novelty of this area of study, no long-term studies exist at present in this area. Considering the scarcity of studies in this area, conducting a scoping review was more appropriate as the existing data do not meet the criteria for a meta-analysis. In addition, the normal progression of science/research in physical activity/exercise research focusses on short-term observations and once a trend of efficacy has been demonstrated, long-term studies can be undertaken, hence the need for this scoping review, as a precursor to systematic reviews, to demonstrate the efficacy of short-term interventions before moving onto longer term investigations [42]. In this manner, a systematic review can be supported by preliminary evidence-based scoping review findings.

Traditional dancers and game participants experienced pleasure and enjoyment from these cultural activities, which might be essential in influencing regular PA participation and adherence to achieve long-term health benefits.

This could explain the improved quality of life observed in the included studies [13,24,29,30,33]. Two non-RCT studies demonstrated a direct link between intrinsic motivation for participation, socio-cultural connectedness, physical benefits, and overall quality of life brought by culturally relevant dance and games [19,33]. It has been suggested that intrinsic motivation, consistent energy expenditure balance, and adherence to exercise programs play a vital role in long-term cardiovascular health. Studies have indicated that traditional dance and games can serve as effective exercise interventions for improving CVDs [24,33]. It should be noted that traditional dance and games often involve physical activity where participants use their bodies in ways that promote endurance, strength, agility and balance [11,13,23]. In this regard, by engaging in traditional dance and games activities regularly, indigenous people subsequently improve their cardiovascular health and physical functioning and thus have reduced cardiovascular risk factors. Thus, traditional dance and games are not only ideal alternative PA for improving individuals’ health and well-being but also align well with individual or group preferences [3], are easily accessible, and are culturally appropriate. These are key considerations for participants and practitioners in addressing CVDs.

### 4.1. Implications for Practice

It is important to consider immediate multi-strategy interventions by collaborating with healthcare providers and indigenous communities in LMICs. Incorporating indigenous knowledge gained through engaging communities, and striking a balance between evidence-based medicine, conventional exercise training, and culturally sensitive interventions such as traditional dance and games, might be effective in fostering physical activity among underserved populations. Townsend highlighted the need to prioritize community-led prevention strategies for NCDs [25], to improve CVD outcomes [13], and suggested that incorporating cultural activities [24,37] can enhance physical activity, lifelong participation, reduce health costs [11], and ultimately improve practice. Given that this review addressed different study populations, ages, genders, and multiple cardiovascular health outcomes, it is vital for sports and exercise scientists, exercise trainers, physical activity leaders, clinicians, and other healthcare providers to optimize the most effective health promotion and prevention strategies that are culturally relevant and cost-effective. This could be particularly useful in LMICs where existing traditional educational programs and structures incorporating traditional dance, games, and sport enshrine their deep socio-cultural values and socio-political ideologies.

### 4.2. Implications for Policy

Our review findings demonstrate critical evidence is only available from limited studies on CVDs in LMICs, particularly in Africa, highlighting a significant gap in research. This is concerning given the rising deaths and threats posed by CVDs in these regions, which calls for urgent systematic approaches to address these gaps. To create effective policies and interventions for CVDs in LMICs, it is vital to increase funding targeted for research, to contextualize policy and physical activity guidelines to ensure cultural and socio-economic relevance, and to consider healthcare system and infrastructure differences [41].

Traditional dance and games, as culturally relevant, community-based, physical activity/exercise interventions, should be both theoretically informed and evidence-based, and should be delivered by adequately trained coaches with related skills and knowledge. Furthermore, future research on traditional dance and games should focus on optimising intensity, injury prevention, and clinical or health-related outcomes related to CVD risk. Research should focus on CVD management and prevention strategies, particularly in elderly populations in LMICs. A notable finding of this review was the lack of follow-up observation of participants in the included studies. LMIC policymakers should promote community-led prevention strategies that are culturally relevant and effectively increase education which encourages PA participation and active healthy lifestyles. This must be complemented by strengthening healthcare systems and tailoring to specific contexts to ensure the effective management of CVDs.

### 4.3. Strengths and Limitations

Our scoping review has several strengths. Firstly, it was conducted following the PRISMA-ScR guidelines and JBI methodology, ensuring an in-depth inclusion of study designs and systematic exploration of the literature via databases and other methods. Secondly, databases with rich traditional dance and games literature from LMICs were selected to address the specific questions posed. The comprehensive nature of this review is another strength, as several CVD outcomes, including cardiorespiratory fitness, body composition, blood biomarkers, and physical function, were obtained and synthesized. Additionally, the inclusion of both RCTs and non-RCTs (e.g., cross-sectional and observational studies) from LMICs provides a broader and more accurate understanding of the effects of traditional dance and games interventions. Lastly, incorporating literature across different age groups ensured that this review encompassed the most relevant data regarding the effects of traditional dance and games interventions, which is crucial for highlighting gaps in CVD prevention in LMICs.

However, there are limitations to consider. The inclusion of only English-language publications may have limited the scope, excluding studies in the non-English literature. Additionally, most studies had small sample sizes, which may affect the ability to detect significant differences in the outcomes assessed. Furthermore, nine of the twelve studies lacked follow-up post-intervention, and only one study compared traditional dance and games with conventional exercise programs for older adults, meaning the results cannot be generalized. It is important to note that traditional dance and games have shown limited substantial benefits for cardiovascular health outcomes. The conclusions are inconclusive due to variations in the available data, such as differences in participant ages, exercise intensities, and levels of engagement in specific dances or games. Addressing these limitations will be crucial for generating more reliable empirical data and guiding future research aimed at improving the health of populations in LMICs, particularly among older adults. Delivery of dance and game programs could ultimately improve the management and prevention of CVDs in LMICs.

## 5. Conclusions

Most of the studies failed to examine simultaneous improvements in multiple health outcomes or determinants. The findings of this review indicate that traditional dance and games are feasible alternatives to conventional exercise programs. Further, it provided substantial evidence supporting improvements in cardiovascular health across age groups. These improvements may be associated with better body composition, cardiorespiratory fitness, lipid profiles, and functionality. Moderate-intensity traditional dance and game interventions, which are community-based, culturally relevant, and cost-effective, can serve as an alternative form of physical activity to improve cardiovascular, metabolic, and functional health in the elderly. Additionally, our findings have potential implications for economical and legislative support, as well as changes and reconfigurations in health policy and their subsequent implementation.

### Future Research

Future research should focus on comparisons of traditional activities to conventional exercise programs, which would help assess their relative effectiveness on cardiovascular health outcomes in diverse LMIC settings. Studies should prioritize larger sample sizes, extended follow-up periods, and comparisons with conventional exercise programs to enhance generalizability. Research should investigate the optimal intensity, frequency, and duration of interventions, while also assessing potential injury risks and strategies for injury prevention. Additionally, the influence of cultural factors on participation and adherence to these activities should be examined. A comprehensive understanding of these factors will guide the development of culturally tailored, sustainable physical activity interventions for cardiovascular disease prevention in LMICs.

## Figures and Tables

**Table 1:** Search parameters.

Abbreviation Description		Question Components
P	Population	Children, adolescents, youth, adults, older adults
I	Concept	Association between traditional dance and improved cardiovascular health
C	Context	Low- and middle-Income countries (LMICs)
O	Outcomes	Cardiorespiratory fitness, body composition, blood biomarkers, physical function
S	Study designs	RCT or non-RCT (i.e., cross-sectional/observational)

**Table 2:** Impacts of traditional dance on cardiovascular health across diverse low- and middle-income countries.

Study	Participant Description	Study Aim	Intervention	Outcome and Measures	Key Findings and Conlusions	Limitations
Duarte et al., 2023 [29] Non-RCT Brazil (upper middle income)	N = 26 Women Age = 20–40 years old	To investigate the effect of 12 weeks of rehearsals on cardiorespiratory parameters and body composition in Brazil samba dancers belonging to a first-league samba school.	Brazilian samba dance 12 weeks	Cardiorespiratory parameters Body composition	Samba dance can increase PA levels and positively affect the dancers’ health parameters, *p* < 0.05 ↑ maximal oxygen uptake ↑ oxygen pulse ↑ lean body mass ↓ body fat % ↓ fat mass.	Only one Samba school with a small sample size was investigated. Samba schools practice different samba with different intensities, causing varying effects on cardiorespiratory parameters and body composition. Evaluation of HR measurements during rehearsals and body composition evaluations were not performed with standard tools.
Wang et al., 2023 [30] RCT China (upper middle income)	N = 26 Women Age—not stated	To examine the effects of simplified dance on body composition, cardiorespiratory fitness, and blood lipids in obese older women.	Simplified dance 12 weeks	Anthropometric measures Cardiorespiratory fitness Blood lipids	Simple dance interventions have potential to improve blood composition and aerobic fitness in obese older women, *p* < 0.05 ↑VO_2_ max ↑ high-density lipoprotein (HDL-C) ↓ total cholesterol (TC).	Sample only included women with obesity and results cannot be generalized for other populations including men. Biomechanical evaluations were not performed.
Daca et al., 2023 [24] RCT Mozambique (low income)	N = 57 older women Age: 60–80 years old	To compare the effects of Conventional Exercise Program (CEP) and Culturally Relevant Activities (CRA) on markers of risk factors for cardiovascular diseases, body composition, functional fitness, and self confidence in older women in living in Maputo City, Mozambique.	CEP (stationary cycling, resistance circuit training) CRA (dances and games, i.e., Bobo nhangua, Salt statue, Marrabenta, Xigubo dance) 12 weeks	Body fat, resting blood pressure Blood glycemic, cholesterol, triglycerides, and high-density lipoprotein Physical fitness, self-efficacy, self-esteem	Both have positive effects on biological and psychological health of older women. ↑ cardiorespiratory fitness and ↑ triglycerides ↑ physical fitness ↑ functional fitness *p* < 0.05 ↑improved quality of life.	Relatively low sample size and only females. There was no control to training intensity and volume in CRA sessions—no equipment was used on individuals compared to CEP ergometric equipment. The self-efficacy tool (McAuley questionnaire) was not sensitive enough to detect changes. No follow-up post-intervention.
Ajala et al., 2020 [31] RCT Nigeria (lower middle income)	N = 30 Obese men Age: 18–22 years old	To assess the effect of aerobic dance training on selected health related fitness variables among obese men.	Aerobic dance 8 weeks	Cardiorespiratory endurance Body composition	Aerobic dance improved body composition and cardiorespiratory endurance, *p* < 0.05.	Not stated
Gebretensay et al., 2018 [32] RCT Ethiopia (low income)	N = 100 Boys and Girls Age: 15 to 17 years old	To evaluate the effect of Tigray dance on selected physiological variables among high school students.	Traditional dances of Tigray region (Awris, Hura, Kuda, Shediva) 16 weeks	Heart rate Systolic blood pressure DBP	Traditional dance treatment groups showed significant improvement in physiological variables, resting HR, SBP, DBP, *p* < 0.05.	Not stated
Rodrigues-Krause et al., 2018 [13] RCT Brazil (upper middle income)	N = 30 Sedentary women Age: 60–75 years old	To compare the effects of dancing and walking on cardiovascular disease and functionality of older women.	Dancing—several styles 8 weeks	Cardiorespiratory fitness Body composition Lipid profile VO_2_ peak Balance	Clear clinical relevance. ↑ CRF in walking ↑ lower body muscle power in dancing. Increased PA levels were noted. ↑ VO_2_peak ↓ body composition ↑ lipid and inflammatory profile, *p* < 0.05.	Small size sample to detect the differences on a variety of outcomes assessed. No follow-up post-intervention.
Nxumalo et al., 2015 [33] Non-RCT South Africa (upper middle income)	N = 44 Male children Age: 9–10 years old	To investigate the potential influence of the traditional Zulu stick fighting game on health-related physical fitness of prepubescent males.	Indigenous game—Zulu stick fighting 10 weeks	Body composition Cardiovascular fitness Muscle endurance and strength	Significant differences were noted. ↑ cardiovascular fitness ↓ body composition ↑ flexibility No significant change—muscle strength and endurance, *p* < 0.05.	Small sample size limited statistical power of analysis.
Kin et al., 2001 [34] Non-RCT Turkey (upper middle income)	N = 45 Female college students Age: SA = 21.88 ± 2.16; AD = 20.23 ± 0.16; Control = 21.88 ± 1.82 years old	To examine the effects of 8 weeks of step aerobics and aerobic dancing on blood lipids and lipoproteins.	Aerobic Dance 8 weeks	Cardiovascular risk factors Blood lipids Lipoproteins	Step aerobics training had more benefits than aerobic dancing for serum TC, HDL-C levels, and TC: HDL-C ratio. Favourable changes in serum TC levels resulted after aerobic dancing.	The conclusions of the study must be limited to the studied population.

RCT—Randomised control trial, Non-RCT—Non-randomised control trial, PA—Physical activity, CRF—Cardiorespiratory fitness, ↑—increase, ↓—reduction, BP—Blood pressure, CVD—Cardiovascular disease, HR—Heart rate, SBP—systolic blood pressure, DBP—Diastolic blood pressure, TC—Total Cholesterol, HDL-C—High-Density lipoprotein.

**Table 3:** Impact of traditional games on cardiovascular health across diverse low- and middle-income countries.

Study	Participants	Aim	Intervention	Outcome	Key Findings	Limitations
Manihuruk et al., 2024 [35] Non-RCT Indonesia (upper middle income)	N = 30, Children, Age: 9–11 years old	Measure intensity of Malaysian traditional games.	Traditional game of peach piring, 7 weeks	Body measures, HR, METs, vector magnitude	Three games met MVPA standards for steps, HR, vector magnitude.	Upper body motions not well assessed, findings limited to northern regions.
Yulia et al., 2021 [36] Non-RCT, Indonesia (upper middle income)	N = 72, Students, Age: Not stated	To check the effects of nutrition education and Javanese games on lipids in overweight children.	Traditional games (Galasin, ucing, kup, luncat tinggi and sapintrong/jimping rope), Nutrition education, 12 weeks	Cholesterol, triglycerides, lipid profiles (LDL-C, HDL-C)	Games lowered cholesterol and triglycerides but did not improve lipid profiles, *p* > 0.05.	Not stated.
Malik et al., 2021 [19] Non-RCT, Malaysia (upper middle income)	N = 600 (300 boys, 300 girls), Age: 10.2 ± 0.8 years old	To measure exercise intensity and enjoyment.	Five traditional games (poison ball, runner and tagger, Police and Thief, build-destroy-rebuild a pyramid)	Body measures, HR, METs, enjoyment response	Games supported MVPA and boosted enjoyment, aiding health and exercise habits, *p* > 0.05.	HR results were post-game only, limited to 5 games.
Rauber et al., 2014 [37] Non-RCT, Brazil (upper middle income)	N = 16 (8 boys, 8 girls), Age: 9–10 years old	To check if BP stress reactions drop after play vs sedentary activity.	Three traditional games (run and catch, dodge ball, and capture the flag)	Post-exercise BP, SBP, DBP	Games reduced BP stress response after one session, *p* ≤ 0.05.	Small sample, gender comparison not possible, short monitoring time, genetics and ethnicity not considered.

RCT—Randomised control trial, Non-RCT—Non-randomised control trial, PA—Physical activity, CRF—Cardiorespiratory fitness, ↑—increase, ↓—reduction, BP—Blood pressure, CVD—Cardiovascular disease, HR—Heart rate, SBP—systolic blood pressure, DBP—Diastolic blood pressure, HEP—Post-exercise hypotension, MEs—metabolic equivalents, MVPA—moderate-to-vigorous PA.

**Table 4:** Countries investigated and the associated Gross National Income per capita classification and Gross Domestic Product, 2023 [38].

Country	Atlas GNI per Capita (Billions of USD)	GDP (Millions of USD)
Brazil	Upper-middle income	2,173,666
China	Upper-middle income	17,794,783
Ethiopia	Low income	163,698
Indonesia	Upper-middle income	1,371,171
Malaysia	Upper-middle income	399,705
Mozambique	Low income	20,954
Nigeria	Lower-middle income	363,846
South Africa	Upper-middle income	380,699
Turkey	Upper-middle income	1,118,253

GNI—Gross National Income per capita (total wealth of a country). Low income, USD ≤ 1135; lower-middle income, USD 1136—4465; upper-middle income, USD 4466–13,845; high income USD > 13,845–4515. GDP—Gross Domestic Product (total market value of goods and services produced by a country within a specific period).

## Data Availability

The data presented in this study are available on request by the corresponding author.
